# Lightweight Deep Learning Classification Model for Identifying Low-Resolution CT Images of Lung Cancer

**DOI:** 10.1155/2022/3836539

**Published:** 2022-08-30

**Authors:** Shanmugasundaram Marappan, Muhammad Danish Mujib, Adnan Ahmed Siddiqui, Abdul Aziz, Samiullah Khan, Mahesh Singh

**Affiliations:** ^1^Department of Computer Science, College of Computer Science & Information Technology, Jazan University, Jizan, Saudi Arabia; ^2^Department of Biomedical Engineering, NED University of Engineering and Technology, Karachi, Pakistan; ^3^Department of Computing, FEST Hamdard University, Karachi, Pakistan; ^4^Department of Software Engineering, National University of Computer and Emerging Sciences, Karachi, Pakistan; ^5^Department of Maths, Stats & Computer Science, The University of Agriculture Peshawar, Peshawar, Pakistan; ^6^Kebri Dehar University, Kebri Dahar, Ethiopia

## Abstract

With an astounding five million fatal cases every year, lung cancer is among the leading causes of mortality worldwide for both men and women. The diagnosis of lung illnesses can benefit from the information a computed tomography (CT) scan can offer. The major goals of this study are to diagnose lung cancer and its seriousness and to identify malignant lung nodules from the provided input lung picture. This paper applies unique deep learning techniques to identify the exact location of the malignant lung nodules. Using a DenseNet model, mixed ground glass is analyzed in low-dose, low-resolution CT scan images of nodules (mGGNs) with a slice thickness of 5 mm in this study. This was done to categorize and identify many histological subtypes of lung cancer. Low-resolution CT scans are used to pathologically classify invasive adenocarcinoma (IAC) and minimally invasive adenocarcinoma (MIA). 105 low-resolution CT images with 5 mm thick slices from 105 patients at Lishui Central Hospital were selected. To detect and distinguish, IAC and MIA, extend and enhance deep learning two- and three-dimensional DenseNet models are used. The two-dimensional DenseNet model was shown to perform much better than the three-dimensional DenseNet model in terms of classification accuracy (76.67%), sensitivity (63.3%), specificity (100%), and area under the receiver operating characteristic curve (0.88). Finding the histological subtypes of persons with lung cancer should aid doctors in making a more precise diagnosis, even if the image quality is not outstanding.

## 1. Introduction

CT or CAT scanning, often known as computed tomography, is a noninvasive testing method. With the use of computer technology and a specialized sort of X-ray, a CT scan may provide cross-sectional pictures (slices) of soft tissue, organs, bone, and blood arteries in any part of the body. The development of CT lung cancer screening has transformed medical imaging by delivering more thorough data than traditional X-rays and, eventually, better patient care. Chest X-ray, low-radiation chest computed tomography (CT), and standard radiation chest CT are imaging techniques used to assess the lungs. Due to its ability to identify cancer with greater sensitivity than X-rays while exposing patients to less radiation than regular chest CT, low-radiation-dose CT is suitable for cancer screening. Lung cancer is the most common type of cancer that kills people around the world, with an 18% chance of survival after 5 years [1]. Pulmonary nodules are one of the early signs of lung cancer, and as low-dose CT scanning and lung cancer screening become more common, they are increasingly used. Pulmonary nodules can be divided into solid nodules, pure ground-glass nodules (pGGNs), and mixed ground-glass nodules (pGGNs) based on whether or not they have solid parts [2]. Mixed ground-glass nodules are more likely to be cancerous than the other two types of lung nodules [3, 4].

In 2015, the World Health Organization (WHO) put lung adenocarcinoma into a category and said that it is a type of lung cancer. The pathology could be invasive adenocarcinoma (IAC) or minimally invasive adenocarcinoma (MIA) [5–7]. The imaging signs are called mGGNs, and the pathology could be invasive adenocarcinoma (IAC) or minimally invasive adenocarcinoma (MIA) (MIA). Even though both MIA and IAC are cancerous growths, the 5-year survival rates for each surgery are different. Patients with MIA who have complete surgical resection have a 5-year survival rate of 100% or very close to 100%. Recent data suggest that sublobar resection may be a good option for these lesions. Patients with IAC who need sublobar resection have a 74.6 percent 5-year survival rate [7–10]. It is clear that using CT imaging to classify and find these two histological subtypes of lung cancer has important clinical implications.

The classification of lung adenocarcinomas includes a relatively recent category called minimally invasive adenocarcinoma (MIA). This group of lesions includes tiny, solitary adenocarcinomas that are less than 3 cm (or less than 30 mm) in size and have either purely lepidic growth or mostly lepidic growth with less than 5 mm of stromal invasion. Localized lung adenocarcinomas that are less invasive have a diameter of less than 3 cm, a prominent lepidic growth pattern, cancerous cells along alveolar structures, and a stromal invasion of less than 5 mm. These lesions should not have pleural, lymphatic, or vascular invasion. Nonmucinous histopathological subtypes include the following three: Mucinous: goblet cell and mucus-secreting, out of which mucus-secreting is frequently multicentric, uncommon and mixed, and is by far the most prevalent subtype. Similar to adenocarcinoma in situ, lung cancer that is only marginally invasive cannot be accurately identified without a complete histologic sample of the tumor.

Invasive apocrine carcinoma (IAC) is a kind of breast cancer that has a morphologic look similar to apocrine sweat glands in at least 90% of tumour cells and is tightly associated with androgen receptor expression. Molecular research suggests that invasive apocrine carcinomas are a unique subtype of breast cancer. IAC is distinguished by a pattern of gene expression that is substantially influenced by AR expression. AR might be a therapeutic target. IAC did not cluster with the basal-like group despite being negative for ER, PR, and HER2. About half of the patients tested negative for ER and PR but positive for HER2. Tumors have a high degree of overlap with the HER2 group, as determined by intrinsic gene categorization. Data from gene expression reveal a relationship between HER2 signalling and the molecular apocrine phenotype. The gene signature contains elevated expression of several genes involved in metabolism.

Pulmonary nodules, which are groups of abnormal cells in the lungs and may be the first signs of lung cancer, are found on CT imaging. Prior to the onset of lung cancer's clinical symptoms, these nodules are frequently identifiable on CT. It has been demonstrated that early pulmonary nodule diagnosis using CT screens improves survival compared to those who do not have a lung CT scan. Although pulmonary nodules are common, not all of them are malignant. In actuality, the majority of nodules are not malignant and are brought on by scar tissue from a previous lung infection. Small nodules are routinely seen during computed tomography screening but are subsequently shown to be benign. Additional diagnostic procedures will be advised if a nodule raises cancer suspicion.

The authors [11] investigated the efficacy of computer-aided quantitative analysis in the differential diagnosis of several invasive lung adenocarcinomas and assessed the repeatability of computer-aided quantitative measurement. Quantitative analysis of preoperative CT imaging signs was discovered by the authors [12]. It is essential to identify adenocarcinoma in situ (AIS) and malignant adenocarcinoma in situ (MIA) from pulmonary ground-glass nodule IAC. The authors reviewed the CT differential diagnosis of MIA and IAC exhibited as pGGNs [13]. When pGGNs lesions are accompanied by lobulation signs, speculative symptoms, or air bronchus signs, CT scans of the chest with a thickness of 5 mm have shown that the bronchus in pGGNs is twisted and dilated. The vascular bundle sign is more likely to be IAC large. These quantitative analysis studies suggest that CT imaging has a discriminative value for classifying lung adenocarcinoma histological subtypes.

Many hospitals in China still use thick-slice CT scans for lung cancer screening. 5 mm thick CT scans are the most conventional scanning methods. The dose and intensity of X-rays are lower, and the damage to the human body is more minor. However, 5 mm thick CT scan image resolution is low, texture information is scarce, and it is challenging to classify and identify histological subtypes of lung adenocarcinoma. With computer technology and extensive data development, deep learning has developed rapidly in recent years. The classification and identification of lung adenocarcinoma histological subtypes in slice-thick CT images provide the possibility. Profound knowledge can be applied to the detection, segmentation, and diagnosis of lung nodules in CT images, assisting radiologists in their work, reducing the rate of missed diagnosis, and increasing the diagnosis accuracy rate. Many researchers have devoted themselves to using deep learning technology to classify benign and malignant pulmonary nodules [14, 15]. However, there are still few studies on the classification of lung adenocarcinoma histological subtypes. As a result, the goal of this work was to use deep learning technology to categorize and detect lung cancer histological subtypes using low-dose CT scan pictures with a 5 mm slice thickness.

Deep learning has found considerable success in the image processing field in recent years due to its superior learning capabilities. Deep learning, as shown by DenseNet, is increasingly being used in medical imaging, with promising findings in clinically aided classification, identification, detection, and segmentation of benign and malignant tumours, brain function, cardiovascular illnesses, and other significant diseases. DenseNet efficiently uses high-level data to rediscover innovative features at the bottom layer, improving feature transfer across the network and enabling increased feature reuse, resulting in a reduction in the number of parameters. The model relies on DenseNet and uses the transitional dense projection technique to gather three-dimensional data about pulmonary nodules. It then trains the infrastructure of focal loss to facilitate it to concentrate on learning the challenging rectified lung nodules with promising experimental outcomes. This study proposes a method based on the deep learning DenseNet [16] model to distinguish IAC and MIA that appear as mGGNs on images from low-resolution (5 mm slice thickness) thick CT screening and explores a deep learning method. The differential diagnosis of IAC and MIA is expected to assist radiologists in predicting and guiding the histological subtypes of patients with lung adenocarcinoma in lung cancer screening and providing a basis for selecting clinical treatment methods and prognosis judgment.

## 2. Materials and Methods

### 2.1. Data Sources

**Figure 1:**
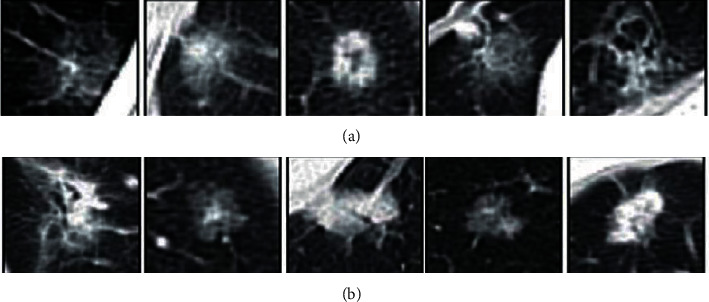
CT images with confirmed IAC and MIA [2]. (a) IAC. (b) MIA.

Between January 2015 and December 2016, 105 patients' chest CT scans with a 5 mm slice thickness were reviewed retrospectively, and 105 CT pictures with mixed ground-glass nodules were chosen. All ground-glass nodules were pathologically identified, with IAC and MIA being confirmed in 71 and 34 cases, respectively.  Table 1 contains the specific comparable imaging findings, which are shown in Figure 1. It is easy to make errors assessing the histological subtypes of mGGNs lung adenocarcinoma when clinicians forecast pathological outcomes from imaging. Computer-aided diagnostic technology, especially the deep learning approach, may support clinicians in giving reference material for identifying benign and malignant lung nodules, according to recent research [14, 15]. To categorize and identify the IAC and MIA that appear as mGGNs on the picture, the deep learning DenseNet model is used.

### 2.2. Scanning Instruments and Methods

Siemens Force dual-source 96-slice CT scanner with 120 kV tube voltage (Germany), for lung imaging, Br40 Kernel, and ADMIRE level 3 were employed for window reconstruction in the mediastinal area. First, a regular scan was carried out. Then, according to a 1.5 mL/kg dosage injection, an iodixanol (containing iodine 320 mg/mL) contrast agent was delivered into the cubital vein at a rate of 3.0 mL/s. The arterial phase scan was delayed by 28 seconds, and the delayed phase scan was done 30 seconds after the arterial phase scan ended.

### 2.3. Data Preprocessing

**Figure 2:**
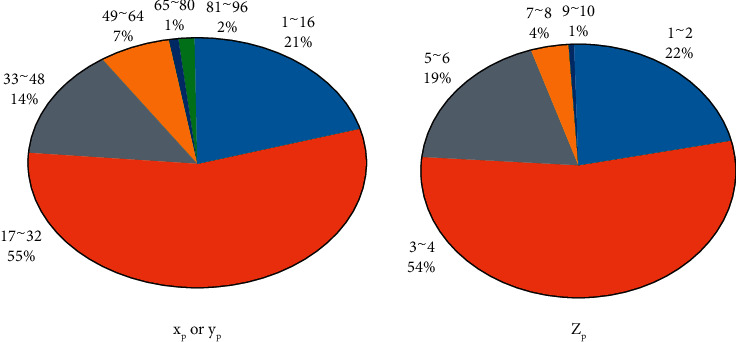
Distribution of the actual number of pixels occupied by the diameter of the long axis of the nodule [4].

Data annotation was performed on the obtained low-dose CT images with a thickness of 5 mm, and the localization of the center point ([xnod, ynod, znod]) of mGGNs was completed by two radiologists who had been engaged in the imaging diagnosis of chest diseases for more than five years. Nodules were obtained. After the three-dimensional coordinate point of the center, the size distribution of the long axis diameter of mGGNs is used to determine the size of the nodule sample required for the experiment. The specific method is as follows: the long axis diameter (unit: mm) and the image pixel spacing ([xps, yes, zps], unit mm/pixel) to get the actual number of pixels occupied by the diameter of the long axis of the nodule ([*xp*, *yp*, *zp*], unit: pixel); see Figure 2 for details. The algorithm's goal is to mimic the display-style *x*^2^ + *y*^2^ = *r*^2^ curve using pixels. To put it differently, every pixel should be around the same distance from the center. At each step, the route is lengthened by selecting the next pixel that maximizes the display-style *x*^2^ + *y*^2^ but fulfils display-style *x*^2^ + *y*^2^ ≤ *r*^2^ at the same time. Since the candidate pixels are close together, just bit shifts and additions are needed to calculate the latter statement. However, a simplification may be used to better comprehend the bit shift. Remember that a binary number's left bit shift is equivalent to a multiplication by two. Therefore, a left bit shift only results in the diameter, which is equal to the radius divided by two. By breaking things down into simple stages and employing a recursive calculation of the quadratic terms from the previous iterations, it is possible to once more avoid the frequent calculations of squares in the circle equation, trigonometric expressions, and square roots. Among them, the range of *xp* (or *yp*) is 9∼92, 64 and below, a total of 102, accounting for 97.14%; *zp* ranges from 1 to 10, 6 and below, a total of 100, accounting for 95.24%. Therefore, in each mGGN image, Two Dimensional sample with [xnod, ynod] as the center and 64 × 64 as the size are used to extract the ROI (region of interest) nodule, Three Dimensional pieces with [xnod, ynod, znod] as the center and 64 × 64 × 6 as the size are used to remove the VOI (volume of interest) nodule. To take advantage of the nodule background information, images around the lesion were preserved. 105 nodule samples (71 IAC samples and 34 MIA samples) were obtained for both ROI and VOI using 5-fold in the cross-validation experiment, 68 samples (34 IAC and 34 MIA samples) were randomly selected as the dataset during each investigation, and 56 nodule samples (28 IAC and 28 MIA samples) were randomly selected from them. The training set is formed, and the remaining 12 nodule samples are used as the test set.

### 2.4. Data Extension

To create a unique collection of fields, common data extensions are needed. A subset or segment is produced from an available data extension using filtered data extensions. You can choose subscribers at random from a data source extension using random data extensions. In the training set, only the translation, rotation, and flip of the image are performed, and the size and quality of the sample data are not changed. The specific method is as follows: The cross section of the nodule sample is used as the benchmark, and the four directions of 1 are the step size, [1, 5] is the pixel range for translation; the nodule samples are rotated with a step size of 30°; the nodule samples are flipped horizontally and vertically. After these are completed, the number of training sets can be expanded to the original 33 times. This study did not use data expansion in the test set, because expansion is a generic idea that may be employed with many learning strategies and various situations. The data characteristics do not have to create an object similarity that is extremely consistent with the interactions, and the learning technique does not have to provide particularly precise predictions for all object pairings.

### 2.5. DenseNet Convolutional Neural Network

The authors of [16] proposed a new convolutional neural network, DenseNet, mainly consisting of two components: a dense block and a transition layer. In each thick block, the output *xl* of layer *l* is satisfying (1), and the nonlinear transfer function *Hl* () between layers includes three consecutive operations: batch normalization, linear rectification function, and 3 × 3 convolution (Conv3 × 3). The hyperparameter *k* is defined as the growth rate. If *I*_l_ () produces *k* feature maps, then layer *l* will have *k*0 + *k* × (l − 1) feature map inputs (*k*0 is the number of channels of the first input layer). This will result in each layer. There are too many inputs, so Conv1 × 1 is introduced as a bottleneck layer before Conv3 × 3 of each tight block to reduce the number of feature map inputs. Every two tight blocks are connected by a neck layer, changing the size of the feature map by convolution and pooling operations:

(1) $$\begin{array}{c} y_{1}=I_{1}=\left(\left[y_{0},y_{1},\ldots ,y_{l-1}\right]\right). \end{array}$$

### 2.6. Two-Dimensional DenseNet and Three-Dimensional DenseNet Models

**Figure 3:**
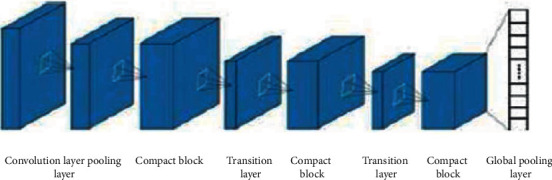
DenseNet basic network model structure.

**Figure 4:**
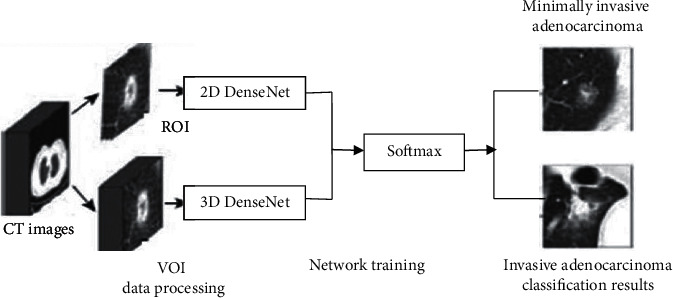
Two-dimensional and three-dimensional DenseNet network model experimental process.

To classify and identify the IAC and MIA that appear as mGGNs on CT images, the DenseNet basic network model is firstly constructed, as shown in Figure 3. Then, according to the characteristics of CT images with a thickness of 5 mm, a specific network is designed based on the DenseNet basic network model. See Figure 4 and Table 2 for details.

In the two-dimensional DenseNet model, the convolutional layer adopts 7 × 7 convolution (Conv7 × 7) and outputs 2k feature maps, while the remaining layers only produce *k* feature maps (*k* = 14) and a global pooling layer. All 3 × 3 pooling (Pool3 × 3) operations are used; considering that the input ROI size is 64 × 64, only three tight blocks are set, and each tight block contains Lblock convolution combinations (Conv1 × 1 + Conv3 × 3); the Lblocks of these three compact blocks are 6, 12, and 24, respectively; between every two close blocks, the transition layers composed of Conv1 × 1 and Pool2 × 2 are used to connect.

All convolution operations have a stride of 1 and zero padding; all pooling operations have a stride of 2 and no zero padding. The difference between the three-dimensional DenseNet model and the two-dimensional DenseNet model is that the input consists of ROI nodules, two-dimensional samples replaced with VOI nodule, and three-dimensional samples, and the ll convolution and pooling sizes are changed from two-dimensional to three-dimensional (e.g., convolution size in convolutional layers is changed from Conv7 × 7 to Conv7 × 7 × 7). After the model obtains the output of the global pooling layer, a Softmax layer is connected at the end, and the Adam optimizer is used to perform the gradient descent algorithm in the process of network training to find the network parameters that minimize the error function.

To avoid the overfitting issue, each convolution operation (excluding the convolutional layer) is followed by a dropout [17] operation. The initial network learning rate is set at 0.005, with 200 iterations (epochs) overall. The learning rate is modified twice throughout the network training procedure. The learning rate is decreased to 10% of the original learning rate after completing 50% of the total number of iterations; after completing 75% of the total number of iterations, the learning rate is reduced to 1%.

The entire experiment was run on an Intel^(R)^ Xeon^(R)^ CPU machine with 32 GB of memory and an NVIDIA GTX-1080Ti GPU (11 GB of memory) to accelerate using Python 3.5, based on the TensorFlow v1.1.0 [18] environment.

The 3D technique performs better than the 2D technique for the segmentation of CT scans. We get dice scores of 79% and 73% for the 3D and 2D techniques, respectively. The 3D technique results in a 5x reduction in the inference time compared to the 2D technique. Results also show that the area plots predicted by the 3D models are more similar to the ground truth than those predicted by the 2D model. Image segmentation is important in a variety of medical imaging applications because it aids in the segmentation of regions of interest. Deep learning techniques for semantic segmentation of medical data have been widely utilized. In recent years, 3D models have been used as predictive algorithms for 3D medical picture data, in addition to 2D neural network architectures. When segmenting CT images, the 3D method works higher than the 2D method. For the 3D and 2D approaches, we receive dice scores of 79 and 73 percent, respectively. In comparison to the 2D methodology, the method reduces the inference time by a factor of 5. From the results, the area plots projected by the models are more accurate than those estimated by the 2D model.

## 3. Experimental Results

### 3.1. Performance Comparison of Two-Dimensional DenseNet and Three-Dimensional DenseNet Models

**Figure 5:**
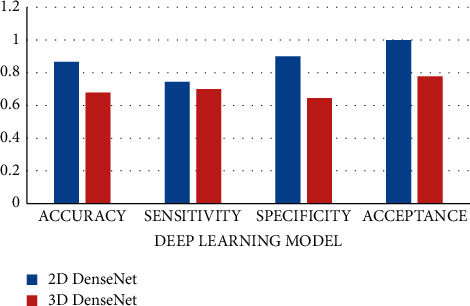
Performance comparison of two-dimensional and three-dimensional DenseNet network models.

The experiment adopts 5-fold cross-validation, with MIA as the positive class and IAC as the negative class, using the ROI and VOI training sets to test the two-dimensional DenseNet and three-dimensional DenseNet models, respectively, set for accuracy, sensitivity, specificity, and acceptance. The two-dimensional DenseNet and three-dimensional DenseNet network models are quantitatively evaluated in terms of the area under the receiver operating characteristic curve (AUC) in four aspects. As shown in Figure 5 and Table 3, the performance of two-dimensional DenseNet model is significant and it is higher than that of the three-dimensional DenseNet model; the former has four performance indicators more elevated than the latter. Although the accuracy of two-dimensional DenseNet is only 76.67%, its AUC value is as high as 0.8889. Literature [19] shows that the accuracy is based on better truncation. The AUC is calculated based on all possible cutoff values, a more robust measurement method than the accuracy. It can be seen that the two-dimensional DenseNet model can effectively perform the IAC and MIA that appear as mGGNs on thick CT images. In addition, the high specificity (90.00%) of two-dimensional DenseNet indicates that the misdiagnosis rate (the probability of classifying MIA as IAC in the case of misclassification) using this deep learning model is low.

### 3.2. Two-Dimensional DenseNet Parameter Adjustment

The parameters of the deep learning network model directly determine its performance. In this section, the bottleneck layer abstention and data expansion are adjusted to explore the impact of these parameters on the two-dimensional DenseNet model. The purpose of designing the bottleneck layer is to reduce the number of feature maps; the abstention is to improve the convergence speed of the two-dimensional DenseNet model. Data expansion can obtain more training data from the limited clinical dataset. These three parameters significantly impact the performance of the two-dimensional DenseNet model, as shown in Table 4. In the table, as is the accuracy, *e* is the sensitivity, *p* is the specificity, and AUC is the area under the curve. The bottleneck layer has the most severe impact.

Under the condition of using bottleneck layer, dropout, and data augmentation, the two-dimensional DenseNet model achieves the best performance in IAC and MIA classification discrimination of mGGNs. To compare the performance with other deep learning network models, this study selects four other two-dimensional deep learning networks (LeNet AlexNet, Agile CNN [15], and Multichannel CNN [14] as controls). LeNet and AlexNet are more classic convolutional neural networks. Agile CNN and Multichannel CNN are new convolutional neural network models proposed in 2018 to classify and identify benign and malignant pulmonary nodules. As can be seen from Table 5, the LeNet model's sensitivity is slightly higher than that of the two-dimensional DenseNet (3.34%). Still, the specificity of the LeNet model is significantly lower than that of the two-dimensional DenseNet (36.67%). In addition, the performance indicators of the two-dimensional DenseNet model are higher than those of other deep learning network models.

## 4. Results and Discussion

The experimental findings reveal that the two-dimensional DenseNet model outperforms the three-dimensional DenseNet model by a substantial margin (see Figure 5). This might be attributed to MIA and IAC having comparable image characteristics, such as mGGNs on the image, resulting in three-dimensional nodule samples with a thickness of 5 mm. As a result, there is greater confusion; nonetheless, the three-dimensional DenseNet model has more parameters to train than the two-dimensional DenseNet model, and the overfitting issue may be more severe. The two-dimensional DenseNet network model outperforms the other four two-dimensional deep learning network models (LeNet, AlexNet, Agile CNN, and Multichannel CNN). This demonstrates that deep learning approaches, particularly the two-dimensional DenseNet model, may be used to categorize and identify lung cancer histological subtypes from thick-slice CT scans. From low-dose CT scan pictures, doctors give a way of analyzing and identifying lung cancer kinds.

**Figure 6:**
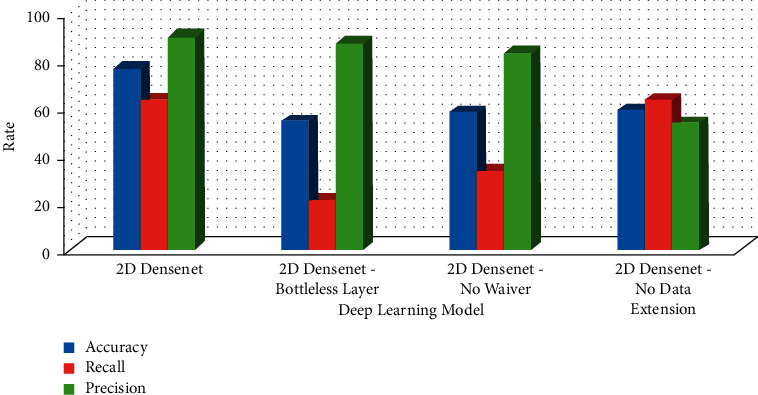
Confusion element of different parameters of the two-dimensional DenseNet model.

**Figure 7:**
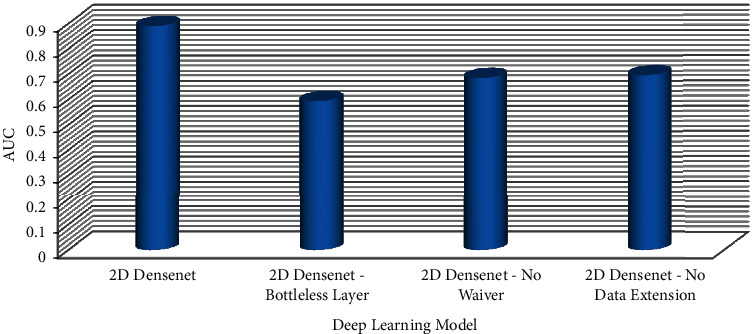
AUC of different parameters of the two-dimensional DenseNet model.

In addition, earlier research looked at using radionics to classify and identify histological subtypes of lung cancer using traditional CT scan pictures. The sequential forward selection (SFS) technique chose 49 optimum picture groups after feature extraction [20]. The ultimate classification accuracy was 70.00 percent (76.67 percent) using a support vector machine (SVM). The two-dimensional DenseNet deep learning algorithm outperformed radionics methods in a CT image classification experiment to predict the histological subtype of lung cancer.  Figure 6 shows the confusion element of different parameters of two-dimensional DenseNet model.  Figure 7 shows the AUC of different parameters of two-dimensional DenseNet model.

**Figure 8:**
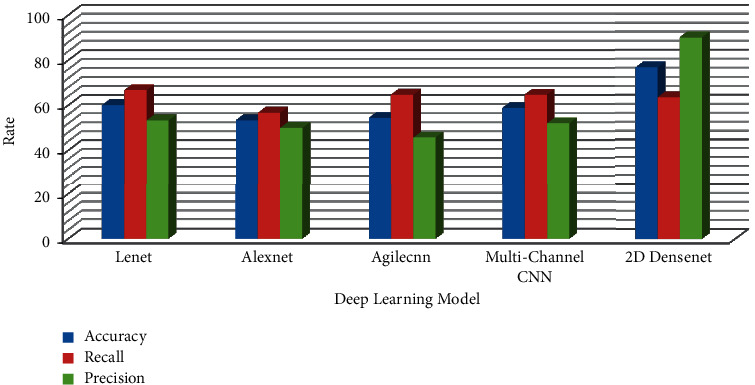
Performance comparison of confusion element of the proposed model with other deep learning network models.

**Figure 9:**
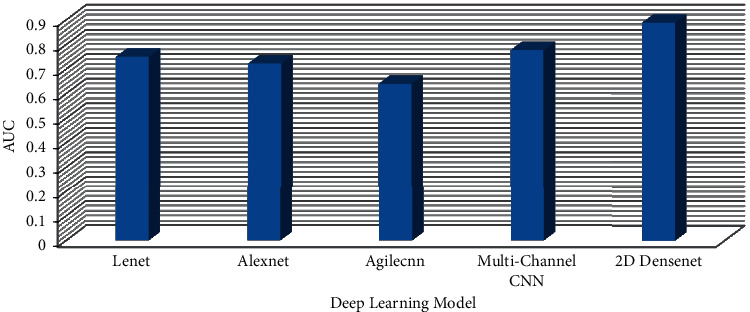
Performance comparison of AUC of the proposed model with other deep learning network models.

Pulmonary nodules are mass formations that range in size from 3 to 30 mm in diameter. Because thick-slice CT scans have poor resolution and limited texture information, a lot of lung nodule information may be lost between slices, posing substantial hurdles in training three-dimensional deep learning networks. As a result, the emphasis of this research is on using a two-dimensional deep learning network model to classify lung cancer in low-resolution CT scans with a slice thickness of 5 mm. Furthermore, ROI nodule two-dimensional samples have a much lower number of features than VOI nodule three-dimensional samples. As a result, overfitting is common when employing the two-dimensional deep learning network model. Because the network model can boost feature propagation and efficiently employ parameters to decrease the phenomena of overfitting, the DenseNet model was chosen as the fundamental technique [16]. Deep learning models like ResNet and CliqueNet may be used to categorize lung cancer histological subtypes based on this.  Figure 8 shows the performance comparison of confusion element of proposed model with other deep learning network models.  Figure 9 shows the performance comparison of AUC of the proposed model with other deep learning network models.

There is still a lack of targeted research on the classification and identification of lung adenocarcinoma histological subtypes from CT images of lung nodules using deep learning technology. The reasons vary, and the most significant limitation is the data on lung adenocarcinoma histological subtype's number. Clinical datasets for histological subtypes of lung adenocarcinoma, especially those with pathological findings, are limited compared to published images of lung nodules. Therefore, in this study, image translation, rotation, and flipping were performed by an operation to increase the data volume of training samples, and the experiments show that the data augmentation method can achieve better classification performance (as shown in Table 4). To overcome the problem of limited datasets, more lung adenocarcinomas need to be collected following data or use of transfer learning techniques. In addition, the number of CT images of pulmonary nodules with pathological findings is limited. Still, the number of lung nodules without pathological findings is relatively large. Future work will examine histological subtypes of pulmonary nodules without pathological findings (especially mGGNs)—classification for research, such as using unsupervised learning techniques.

## 5. Conclusion

This paper presented a DenseNet-based deep learning technique for classifying and distinguishing invasive and less invasive lung cancer from low-dose, low-resolution CT images. ROI nodule two-dimensional samples and VOI nodule three-dimensional samples were obtained as experimental datasets after data preparation. The generated two-dimensional DenseNet model has an AUC of 0.888, accuracy of 76.67 percent, sensitivity of 63.33 percent, and specificity of 90.00 percent. The performance of this deep learning network model outperforms that of the three-dimensional DenseNet model and those of numerous other deep learning network models. The findings of the experiments suggest that deep learning technologies, particularly the two-dimensional DenseNet model, may be utilized to categorize and forecast lung cancer histological subtypes. Further studies will look at additional lung adenocarcinoma data transfer learning and unsupervised learning to aid radiation scientists in doing differential diagnoses on low-resolution CT scans.

## Figures and Tables

**Table 1:** Attributes of patients and pulmonary nodules [5].

Attributes	Each category value
Number of patients	33/72/105 (male/female/total)
Patient age	39/84/62 (min/max/avg)
Number of pulmonary nodules (classified by pathological findings)	71/34/105 (IAC/MIA/total)
Number of pulmonary nodules (classified by long axis diameter)	23/57/20/5 (≤1 cm/≤2 cm/≤3 cm/>3 cm)

**Table 2:** Two-dimensional and three-dimensional DenseNet model structure details.

Convolutional layer	Two-dimensional DenseNet	Three-dimensional DenseNet
Pooling layer	7*∗*7	7*∗*7*∗*7

Compact block (1)	3*∗*3	3*∗*3*∗*3

Transition layer (1)	$\left|\begin{array}{c} 1*1\text{conv}\\ 3*3\text{conv} \end{array}\right|*6$	$\left|\begin{array}{c} 1*1*1\text{conv}\\ 3*3*3\text{conv} \end{array}\right|*6$

Compact block (2)	$\left|\begin{array}{c} 1*1\text{conv}\\ 2*2\text{pool} \end{array}\right|$	$\left|\begin{array}{c} 1*1*1\text{conv}\\ 2*2*2\text{pool} \end{array}\right|$

Transition layer (2)	$\left|\begin{array}{c} 1*1\text{conv}\\ 3*3\text{conv} \end{array}\right|*12$	$\left|\begin{array}{c} 1*1*1\text{conv}\\ 3*3*3\text{conv} \end{array}\right|*12$

Compact block (3)	$\left|\begin{array}{c} 1*1\text{conv}\\ 2*2\text{pool} \end{array}\right|$	$\left|\begin{array}{c} 1*1*1\text{conv}\\ 2*2*2\text{pool} \end{array}\right|$

Global pooling layer	$\left|\begin{array}{c} 1*1\text{conv}\\ 3*3\text{conv} \end{array}\right|*24$	$\left|\begin{array}{c} 1*1*1\text{conv}\\ 3*3*3\text{conv} \end{array}\right|*24$

**Table 3:** Performance comparison of two-dimensional and three-dimensional DenseNet network models.

Serial	Two-dimensional DenseNet	Three-dimensional DenseNet
Accuracy	0.8667	0.6777
Sensitivity	0.7444	0.7
Specificity	0.9	0.6444
Acceptance	0.9999	0.7774

**Table 4:** Performance comparison of different parameters of the two-dimensional DenseNet model.

Deep learning network	Accuracy	Recall	Precision	AUC
Two-dimensional DenseNet [5]	76.67	63.33	90	0.8889
Two-dimensional DenseNet-bottleneck layer [8]	54.17 (↓22.5)	20.84 (↓42.49)	87.50 (↓2.50)	0.5926 (↓0.2963)
Two-dimensional DenseNet-no waiver [10]	58.33 (↓18.34)	33.33 (↓30.00)	83.33 (↓6.67)	0.6833 (↓0.205 6)
Two-dimensional DenseNet-no data extension	58.33 (↓18.34)	63.33 (↓0.00)	53.34 (↓36.66)	0.6944 (↓0.1945)

**Table 5:** Performance comparison of the proposed model with other deep learning network models.

Deep learning network	Accuracy	Recall	Precision	AUC
LeNet [5]	60	66.67	53.33	0.75
AlexNet [6]	53.33	56.67	50	0.7222
Agile CNN [6]	54.34	64.67	45.65	0.6389
Multichannel CNN [7]	58.76	64.66	52	0.777
Two-dimensional DenseNet	76.67	63.33	90	0.888

## Data Availability

The data shall be made available upon request to the corresponding authorf.
